# Progress on novel compounds for the treatment of bone metabolism-related disorders: focus on osteoporosis and beyond

**DOI:** 10.3389/fchem.2026.1861883

**Published:** 2026-07-30

**Authors:** Yeqiong Song, Ruixing Zhang, Xinyue Zhang, Jingkai Wang, Jinyang Chen, Li Li

**Affiliations:** 1 Endocrinology Department, Third People’s Hospital of Hangzhou, Hangzhou, China; 2 Zhejiang University, Hangzhou, China; 3 Zhenjiang Fourth Peoples Hospital and Zhenjiang Women and Children’s Hospital, Zhenjiang, China; 4 Second Affiliated Hospital, School of Medicine, Zhejiang University, Hangzhou, China

**Keywords:** bone metabolism, clinical translation, compounds, osteoporosis, PROTACs, RANKL inhibition, senolytics, Wnt signaling

## Abstract

Bone metabolism-related disorders represent a significant global health burden, due to their high prevalence and substantial socioeconomic costs, particularly osteoporosis. Conventional antiresorptive and anabolic agents have well-established clinical utility, such as bisphosphonates, denosumab, teriparatide, and romosozumab. Nevertheless, their long-term clinical use remains limited by safety concerns and inadequate efficacy in a subset of patients. In this review, we highlight recent chemical innovations designed to mechanistically reprogram the skeletal microenvironment and overcome those longstanding barriers. We also analyze how the metabolic coupling signals govern bone bioenergetics, particularly within the Wnt and RANKL axes. We place specific focus on three emerging and transformative strategies: proteolysis-targeting chimeras (PROTACs), bone-targeted senolytics, and direct metabolic modulators. Ultimately, we provide a multidisciplinary roadmap for the development of next-generation therapeutics to achieve durable skeletal restoration in patients with bone metabolic disorders.

## 1 Introduction

**FIGURE 1:**
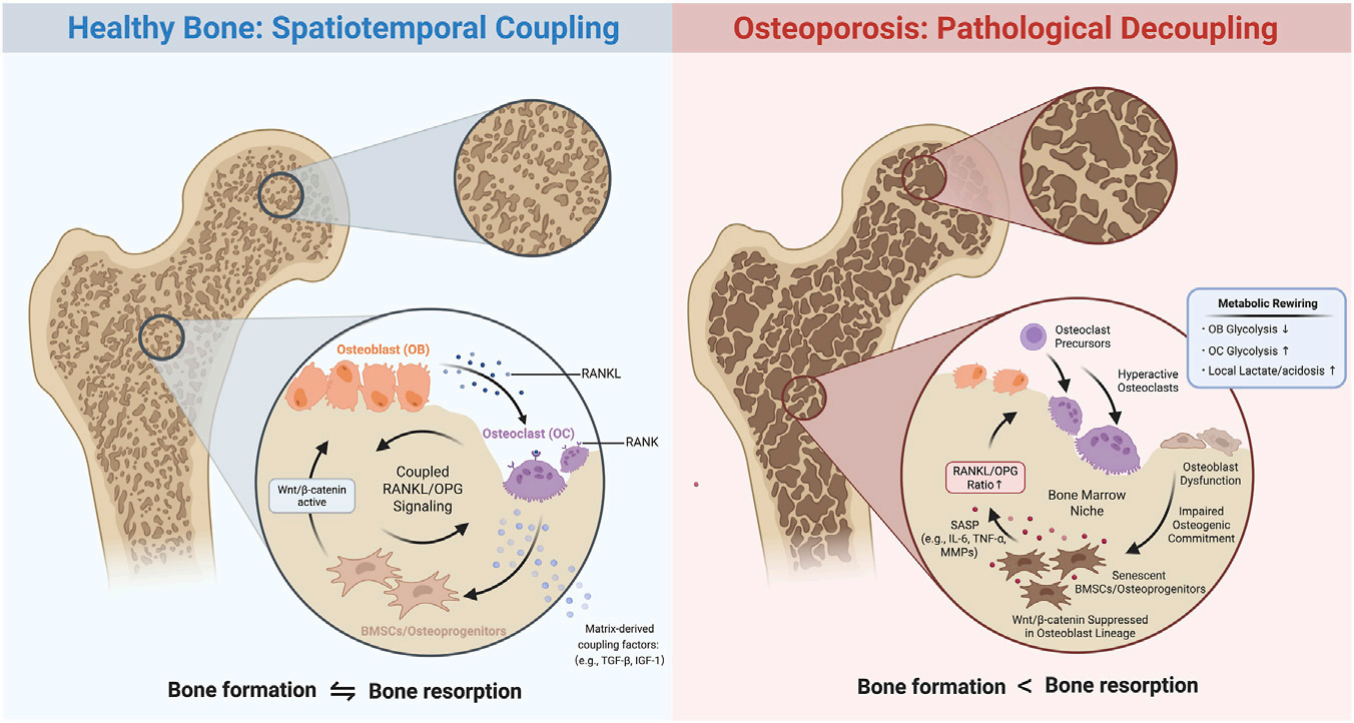
Conceptual framework of physiological coupling and pathological decoupling in bone remodeling. Under physiological conditions, bone remodeling is maintained by the spatiotemporal coupling between osteoclast-mediated bone resorption and osteoblast-mediated bone formation. This balance is supported by coordinated RANKL/RANK/OPG signaling, active Wnt/β-catenin signaling in osteoblast-lineage cells, and the release of matrix-derived coupling factors, such as TGF-β and IGF-1, during bone resorption. In osteoporosis, this coupling is progressively disrupted, leading to trabecular deterioration, suppressed osteogenic Wnt signaling, an increased RANKL/OPG ratio, accumulation of senescent BMSCs, and enhanced SASP output. At the metabolic level, osteoblasts exhibit bioenergetic collapse with reduced glycolytic support, whereas osteoclasts display glycolytic reprogramming and increased local lactate production/acidosis. Together, these changes shift bone remodeling from coupled homeostasis toward pathological decoupling, in which bone formation no longer compensates for excessive bone resorption.

Skeletal integrity depends on the precise spatiotemporal coupling of bone-resorbing osteoclasts and bone-forming osteoblasts (Sims and Martin, 2015). During physiological remodeling, osteoclastic resorption removes aged matrix, and releases matrix-derived coupling factors, notably TGF-β1 and IGF-1 (Crane and Cao, 2013; Crane et al., 2015). Among them, resorption-activated TGF-β1 helps recruit bone marrow mesenchymal stem cells to remodeling sites (Crane et al., 2015; Tang et al., 2009), whereas matrix IGF-1 promotes osteogenic differentiation and subsequent new bone formation (Crane and Cao, 2013; Xian et al., 2012). This coupled remodeling maintains mineral homeostasis, whereas its pathological disruption triggers systemic bone loss and structural failure. Primary osteoporosis affects over 200 million individuals with post-fracture mortality rates reaching 30% (Rodrigo, 2021; Wang and Seibel, 2024). Moreover, a diverse group of high-burden skeletal disorders, notably glucocorticoid-induced osteoporosis (GIOP), osteoarthritis, and tumor-induced bone destruction (TIBD), are recognized as being driven by the pathological decoupling of bone remodeling (Coleman et al., 2020; Steinmetz et al., 2023; Xu et al., 2023). As summarized in Figure 1, these disorders can be viewed as a progressive shift from physiological coupling toward pathological decoupling of the bone remodeling niche (Boyce and Xing, 2008; Fan et al., 2021).

Current antiresorptive and anabolic therapies are well established in clinical practice (Kostenuik et al., 2023; Pavone et al., 2017). However, their long-term use can be limited by cumulative off-target toxicities, and they do not correct upstream metabolic and epigenetic defects (Lee et al., 2017; Lee et al., 2020; Wu J. et al., 2023). To address these limitations, medicinal chemistry has increasingly focused on bone-targeted rejuvenation strategies, including proteolysis-targeting chimeras (PROTACs) for catalytic protein depletion and bone-seeking ligands, such as bisphosphonate conjugates or acidic peptides, for localized drug delivery (Bond and Crews, 2021; Young and Grynpas, 2018). In this review, we examine emerging therapeutics for skeletal microenvironment reprogramming, with a specific focus on three transformative modalities that address the limitations of conventional therapies. We then propose a multidisciplinary roadmap for next-generation therapeutic development, integrating AI-driven discovery and biomarker-guided precision regimens to achieve durable skeletal restoration.

## 2 The conceptual transition in skeletal therapeutics

### 2.1 The bioenergetic basis of bone health

Skeletal homeostasis is tightly coupled to cellular metabolic activity. The biosynthesis of bone matrix by osteoblasts is an exceptionally energy-intensive process, with nearly 80% of the requisite ATP derived from aerobic glycolysis (Lee et al., 2017; Lee et al., 2020). This glycolytic commitment is not incidental, but actively orchestrated by Wnt signaling, which links osteogenic lineage progression to the transcriptional upregulation of glucose transporters and rate-limiting glycolytic enzymes (Bertels et al., 2024; Esen et al., 2013; Lee et al., 2020). Canonically, Wnt ligands engage Frizzled and LRP5/6 co-receptors to stabilize β-catenin and activate osteogenic transcriptional programs (Gaur et al., 2005; Liu et al., 2015; Yue et al., 2018) However, in senile osteoporosis and glucocorticoid-induced models (GIOP), this signal-metabolic axis collapses. Thus, aging osteoblasts are characterized by a marked decline in glycolytic capacity, and this bioenergetic failure compromises matrix mineralization and bone formation (Lee et al., 2017; Weinstein, 2011).

### 2.2 The epigenetic niche: lactate accumulation and histone lactylation

Osteoclasts metabolically reprogram toward hyper-glycolysis, generating massive amounts of lactate to acidify the resorption lacunae (Da et al., 2021). Recent findings reveal that accumulated lactate is not a mere metabolic waste product, but a potent epigenetic driver. Through a novel post-translational modification of histone lactylation (Kla) (Zhang et al., 2019) lactate contributes to transcriptional reprogramming through lysine lactylation-associated chromatin regulation. The latest studies have confirmed that aminoacyl-tRNA synthetases (AARS1 and AARS2) can directly transfer lactate to substrate proteins (Mao et al., 2024; Zong et al., 2024) functioning as lactyltransferases that bypass the traditional lactoyl-CoA intermediate (Li et al., 2024) Although initial findings primarily identified non-histone targets, such as cGAS and p53, recent evidence has expanded this non-canonical catalytic repertoire to include direct histone modifications, such as AARS1-mediated H3K18 lactylation in diabetic nephropathy (Hong et al., 2025). This rapidly evolving paradigm provides a highly plausible mechanistic avenue for investigating histone lactylation in the skeletal microenvironment, highlighting AARS enzymes as novel epigenetic drug targets for bone metabolism.

In the osteoporotic microenvironment, this metabolic collapse extends beyond the osteoblasts themselves, manifesting as a failure of intercellular metabolic crosstalk. For instance, impaired glycolysis in adjacent skeletal endothelial cells severely limits their paracrine secretion of lactate. This microenvironmental deficit directly deprives bone marrow mesenchymal stem cells (BMSCs) of the exogenous lactate required for histone lactylation, thereby epigenetically silencing their osteogenic differentiation potential at the progenitor stage (Wu J. et al., 2023). Concurrently, any remaining extracellular lactate produced by osteoclasts may be less efficiently utilized by mature osteoblasts, due to the thermodynamic constraints of monocarboxylate transporters. Because MCT1 operates as a strict symporter of lactate and protons (H^+^), its cellular uptake efficiency is intrinsically dictated by the transmembrane pH gradient (Felmlee et al., 2020; Halestrap, 2013) The extreme local acidosis, generated by hyper-glycolytic osteoclasts, likely diminishes the favorable gradient. Such extracellular acidosis impairs osteoblast function and prevents matrix mineralization (Brandao-Burch et al., 2005). Conversely, aberrant local lactate accumulation triggers pathological histone lactylation in chondrocytes and drives cartilage degeneration in osteoarthritis (Xia et al., 2024).

### 2.3 The pharmacological bottleneck

The long-term utility of current bone anti-resorptives is constrained by basic stoichiometry. Because traditional inhibitors rely on continuous target occupancy, they merely suppress pathology as long as systemic drug levels remain high. Denosumab exemplifies this vulnerability. Its neutralization of extracellular RANKL acts essentially as a temporary deadlock. Once the denosumab is cleared, the sudden release of this blockade precipitates a violent clinical rebound, characterized by a massive, synchronous wave of osteoclastogenesis (Kim et al., 2022; Kong et al., 2022).

Escaping this stoichiometric trap requires a shift toward ‘event-driven’ pharmacology. Rather than competitively pausing a protein’s function, event-driven agents act catalytically to permanently alter the target state. Because this mechanism uncouples prolonged efficacy from continuous systemic exposure, it fundamentally bypasses the risks of receptor desensitization and rebound. When tethered to bone-homing moieties like bisphosphonates, this catalytic approach allows researchers to confine profound target ablation strictly to active remodeling surfaces, offering a definitive structural workaround to conventional drug limits (Yazdani et al., 2016; Young and Grynpas, 2018).

### 2.4 Mechanistic reprogramming: eradicating the pathogenic niche

Building on the principles of event-driven pharmacology, the field is decisively shifting from reversible signal suppression to the physical eradication of disease drivers (Cromm and Crews, 2017). At the molecular level, this is achieved by coopting the ubiquitin-proteasome system; technologies like molecular glues recruit E3 ligases to force the degradation of traditionally ‘undruggable’ skeletal targets (Schreiber, 2021). This philosophy of definitive clearance translates directly to the cellular level through senolytics. Rather than attempting to block individual inflammatory cascades, these agents target the root of microenvironmental decay by forcing the apoptosis of SASP-secreting cells (Farr et al., 2017). While the severe trabecular toxicity of early pan-senolytics like navitoclax highlighted the dangers of systemic clearance (Sharma et al., 2020), the integration of bone-homing prodrugs and nanoscale delivery systems resolves this barrier. Ultimately, moving from temporary inhibition to the targeted removal of pathogenic elements provides a much more durable framework for skeletal niche restoration.

## 3 Emerging therapeutic targets and novel compounds

**FIGURE 2:**
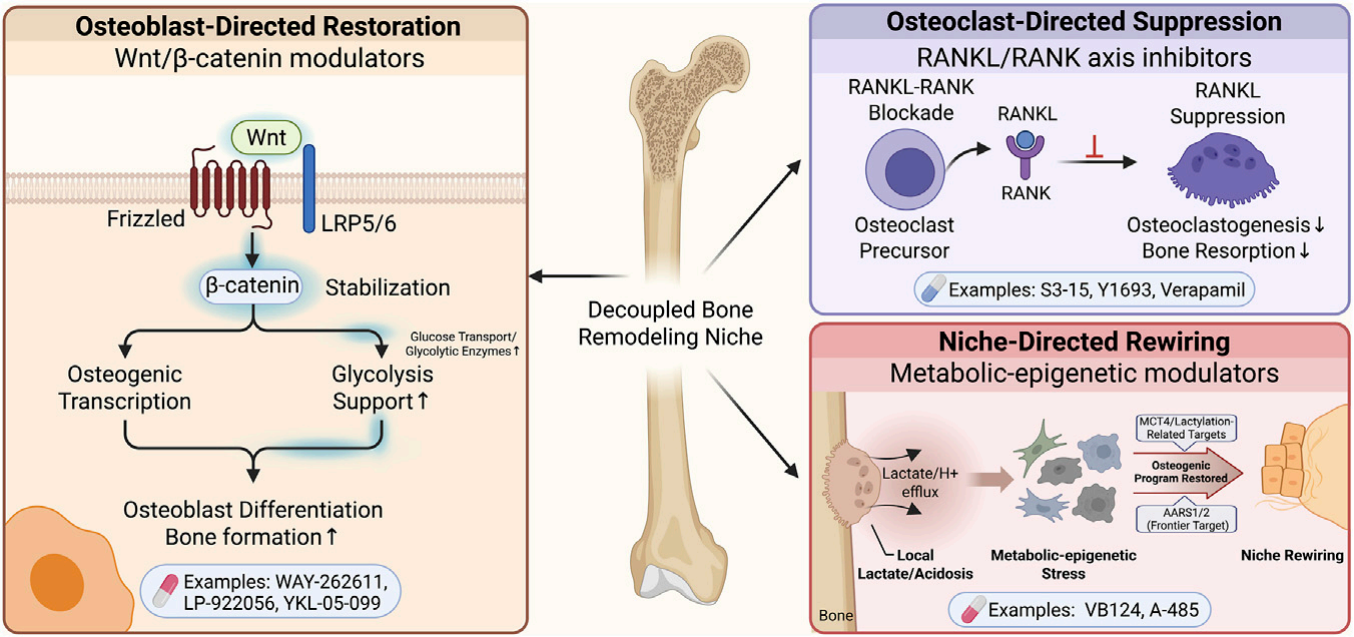
Representative small-molecule strategies for reprogramming the decoupled bone remodeling niche. This schematic summarizes three complementary small-molecule intervention routes for restoring bone remodeling balance. The left panel depicts osteoblast-directed restoration, in which modulation of the Wnt/β-catenin axis stabilizes β-catenin, supports osteogenic transcription and glycolytic metabolism, and promotes osteoblast differentiation and bone formation. The upper-right panel illustrates osteoclast-directed suppression through inhibition of the RANKL/RANK axis, including blockade of the RANKL–RANK interaction and suppression of downstream osteoclastogenic signaling. The lower-right panel shows niche-directed rewiring, in which modulation of osteoclast-associated lactate/acidosis and metabolic–epigenetic stress may help relieve osteogenic suppression within the bone marrow microenvironment. Representative examples include WAY-262611, LP-922056, and YKL-05-099 for Wnt-related modulation; S3-15, Y1693, and verapamil for RANKL/RANK-axis inhibition; and VB124 and A-485 for metabolic–epigenetic regulation, with AARS1/2 indicated as emerging frontier targets.

Current strategies for remodeling the skeletal microenvironment include several directions, such as enhancing Wnt signaling, using targeted degradation, and directly regulating cellular metabolism. To make the following discussion easier to follow, Table 1 provides a condensed overview of representative therapeutic agents, targets, pharmacological logic, and translational status discussed in this review, whereas the full compound-level information and detailed references are provided in Supplementary Table S1. Figure 2 summarizes the key small-molecule strategies for reprogramming the decoupled bone remodeling niche.

**TABLE 1:** Representative therapeutic agents, targets, and pharmacological logic for bone metabolism-related disorders. This condensed table summarizes representative agents, targets, pharmacological logic, and translational status discussed in this review. Full compound-level information and detailed references for the listed compounds and related strategies are provided in Supplementary Table S1.

Therapeutic category	Representative agent/strategy	Primary target(s)	Pharmacological logic	Skeletal translation status/key message
Wnt/β-catenin pathway modulators	WAY-262611	DKK1	Occupancy-driven blockade of an extracellular Wnt antagonist; restores β-catenin signaling	Preclinical Wnt-restorative strategy; relevant to osteogenic deficiency and GIOP.
	LP-922056	NOTUM	Preserves active Wnt ligands by preventing NOTUM-mediated depalmitoleoylation	Preclinical oral NOTUM inhibitor; increases cortical bone mass and strength in models
	Tideglusib	GSK-3β	Non-ATP-competitive irreversible GSK-3β inhibition; stabilizes β-catenin	Repurposing candidate with prior non-skeletal clinical evaluation; skeletal and dental repair evidence remains mainly preclinical
	SKL2001	β-catenin–Axin interaction	Disrupts β-catenin–Axin binding and stabilizes β-catenin without direct GSK-3β blockade	Preclinical osteogenic and bone-defect repair evidence
	YKL-05-099	SIK2/SIK3; CSF1R off-target	Small-molecule PTH-mimetic SIK inhibition; represses SOST and may also limit resorption	Preclinical anabolic and antiresorptive effects; selectivity and dosing remain key issues
	Lorecivivint (SM04690)	CLK2/DYRK1A	Local intra-articular kinase inhibition that modulates Wnt-related transcriptional programs	Clinical-stage OA candidate; repeat-dose data suggest structural benefit despite mixed pain endpoints
RANKL/RANK-axis inhibitors	S3-15	Soluble RANKL	Small-molecule PPI disruption at the RANKL–RANK interface while sparing membrane RANKL function	Preclinical immune-sparing antiresorptive strategy
	Y1693	RANKL trimer/RANK-binding interface	Orally active β-carboline RANKL antagonist; suppresses RANKL-driven osteoclastogenesis	Preclinical OVX bone protection; candidate for antiresorptive optimization
	Verapamil	L-type Ca^2+^ channel/TXNIP–MAPK–NF-κB axis	Repurposed calcium-channel blocker; suppresses TXNIP-mediated MAPK/NF-κB activation and RANKL-driven osteoclastogenesis	Translational/retrospective signal; bone-specific dosing and local pharmacology need validation
Senolytics and senomorphics	Dasatinib + quercetin (D + Q)	Src/ephrin kinases; PI3K/SIRT1/NF-κB-related SCAP networks	Combination senolytic regimen targeting complementary senescent-cell survival pathways	Early clinical test in osteoporosis showed limited overall benefit; biomarker stratification is needed
	Fisetin/PLGA-fisetin	SCAP pathways/anti-apoptotic kinases	Flavonoid-based senolytic activity; nanoformulation improves exposure and osteogenic effects	Preclinical skeletal rejuvenation and osteogenic-lineage support
	SSK1	SA-β-gal-activated prodrug/gemcitabine payload	Senescence-enzyme-responsive prodrug activation selectively releases cytotoxic payload in senescent cells	Selective senolytic activity validated in aged models; bone-specific validation remains limited
​	Ruxolitinib/JAK inhibitors	JAK1/JAK2	Senomorphic suppression of JAK/STAT-driven SASP output	Clinically used JAK inhibitors; skeletal senomorphic translation remains exploratory
Targeted degradation and bone-targeted chemical design	BTK PROTAC compound 23	BTK (via CRBN)	Event-driven catalytic degradation of BTK; suppresses PLCγ2–Ca^2+^–NFATc1 signaling	Preclinical protection against inflammatory alveolar bone loss; supports degrader use in inflammatory skeletal disease
	PZ-15227	Bcl-xL (via CRBN)	Platelet-sparing Bcl-xL degradation by exploiting CRBN availability in senescent cells	Preclinical platelet-sparing senolytic PROTAC; potential relevance to skeletal niche reprogramming, but bone-specific efficacy has not been established
	RANK-targeted LYTAC concept	RANK receptor/lysosomal trafficking receptors	Conceptual lysosome-directed degradation of membrane or extracellular osteoclastogenic drivers	Conceptual strategy only; no RANK-targeted implementation or bone-specific in vivo validation has yet been reported
	BT-Amide	Pyk2 + bisphosphonate bone-targeting group	Bisphosphonate-conjugated heterobifunctional design for hydroxyapatite-directed skeletal enrichment	Proof-of-concept for bone-targeted chemical design; included as a targeting blueprint rather than a PROTAC.
Direct metabolic and epigenetic modulators	VB124	MCT4	Selective blockade of lactate efflux; may limit osteoclast-associated lactate/acidosis while sparing MCT1-dependent handling	Conceptually attractive metabolic modulator; direct osteoporosis validation remains limited
	A-485	p300/CBP	Catalytic inhibition of p300/CBP acetyl/lactyltransferase activity; reduces pathological lactylation signatures	Preclinical OA-relevant evidence; systemic osteoporosis use requires caution due to pleiotropic p300/CBP functions
	AARS1/2-targeted strategies	AARS1 and AARS2	Emerging strategy to uncouple or degrade non-canonical lactyltransferase activity from canonical translation functions	Frontier concept; no bone-optimized inhibitor or degrader has yet been established

AARS, aminoacyl-tRNA synthetase; BTK, Bruton’s tyrosine kinase; CRBN, cereblon; DKK1, Dickkopf-1; GIOP, glucocorticoid-induced osteoporosis; LYTAC, lysosome-targeting chimera; MCT, monocarboxylate transporter; OA, osteoarthritis; OVX, ovariectomy; PPI, protein–protein interaction; PROTAC, proteolysis-targeting chimera; SASP, senescence-associated secretory phenotype; SCAP, senescent-cell anti-apoptotic pathway; SIK, salt-inducible kinase; TXNIP, thioredoxin-interacting protein.

### 3.1 Wnt/β-catenin pathway modulators

The canonical Wnt/β-catenin pathway acts as a master regulator of osteoblast differentiation, and a core metabolic switch coupling osteogenesis with aerobic glycolysis (Chen et al., 2019; Esen et al., 2013; Glass et al., 2005). The anti-sclerostin monoclonal antibody romosozumab has clinically validated the efficacy of Wnt pathway activation. However, its use is limited to a 12-month treatment course, and cardiovascular safety concerns have motivated the development of next-generation small-molecule modulators (Cosman et al., 2016; Langdahl et al., 2017; Saag et al., 2017). These agents aim to achieve intracellular precision, overcome systemic bottlenecks, and extend Wnt-targeted therapy to a broader spectrum of bone pathologies, addressing the stoichiometric limitations of conventional macromolecular biologics.

Physiologically, Wnt ligands bind to Frizzled receptors and LRP5/6 co-receptors on osteoprogenitors, triggering a signaling cascade that stabilizes β-catenin, promotes its nuclear translocation, and activates osteogenic transcriptional programs (Gaur et al., 2005; Liu et al., 2015; Yue et al., 2018). This pathway is tightly negatively regulated by endogenous inhibitors, most notably sclerostin (encoded by SOST) and DKK1, which block LRP5/6 binding to suppress Wnt signaling (Ahn et al., 2011; Li et al., 2005; Li et al., 2006). Below, we categorize emerging small-molecule Wnt modulators by their molecular targets, focusing on their ability to overcome the limitations of conventional therapies, structure-activity relationships (SAR), and translational potential.

#### 3.1.1 Clinically validated Wnt modulators

Prior to romosozumab, teriparatide served as the first-in-class anabolic therapy for severe osteoporosis, acting as a peptide agonist of parathyroid hormone receptor 1 (PTH1R) to stimulate bone formation (Bandeira and Lewiecki, 2022; Neer et al., 2001). Despite its clinical efficacy, teriparatide is limited by daily subcutaneous injection, obligate coupling of bone formation to resorption, and long-term safety constraints (Silva and Bilezikian, 2015). Wnt pathway activation remains empirically validated as clinically viable only through romosozumab. This FDA-approved anti-sclerostin monoclonal antibody has demonstrated a consistent ability to stimulate bone formation while reducing osteoblast-derived RANKL expression (Wu D. et al., 2023). However, its long-term clinical utility is severely restricted by inherent pharmacological and safety limitations. Clinical guidelines stipulate a maximum 12-month treatment period, and a boxed warning of cardiovascular risks requires rigorous patient stratification (Cosman et al., 2016; Langdahl et al., 2017; Saag et al., 2017). Structurally, as a humanized anti-sclerostin monoclonal antibody, romosozumab acts extracellularly by binding and inhibiting sclerostin (Lim and Bolster, 2017). Like most therapeutic antibodies, it does not readily cross the plasma membrane (Singh et al., 2019; Veomett et al., 2013). Accordingly, it is unlikely to directly correct the senescence-associated metabolic defects of the aged bone marrow niche (Lee et al., 2017; 2020; Wu J. et al., 2023). These limitations highlight the urgent clinical need for intracellularly active small-molecule Wnt modulators with improved safety profiles and durable efficacy.

#### 3.1.2 Small molecule Wnt modulators

Small molecule Wnt activators are divided into two mechanistic categories: (1) indirect pathway modulators, which amplify basal Wnt signaling by inhibiting endogenous negative regulators of the cascade; (2) direct pathway agonists, which initiate Wnt signal transduction by directly binding to core positive signaling components. Compared to monoclonal antibody therapeutics, small molecule Wnt agents offer transformative advantages.

##### 3.1.2.1 Extracellular antagonists (DKK1/NOTUM)

Recent advances in chemical biology have identified novel extracellular molecular nodes that effectively bypass bottlenecks in the canonical Wnt signaling cascade, avoiding the need for large biologics.

A promising strategy to target secreted negative regulators has yielded several synthetic small molecule candidates. DKK1, a secreted extracellular inhibitor, attenuates Wnt signaling through competitive binding with LRP5/6. WAY-262611 selectively blocks DKK1 function and promotes β-catenin nuclear translocation. In preclinical models, WAY-262611 significantly increases trabecular bone formation and enhances local osteogenesis, bypassing the need for exogenous Wnt ligands (Li et al., 2022; Tal et al., 2025). Importantly, abrogating DKK1-mediated inhibition of Wnt signaling carries critical translational implications for glucocorticoid-induced osteoporosis (GIOP). High dose glucocorticoids markedly induce DKK1 expression and strongly inhibit osteoblastogenesis (Ohnaka et al., 2004). Thus, WAY-262611 represents a rational countermeasure to rescue the severe osteogenic deficiency in GIOP, filling a critical clinical gap left by standard anti-resorptive therapies. In addition to synthetic compounds, naturally derived agents like the lignan Schisandrin A have shown preclinical activity in GIOP models, indirectly reducing DKK1 transcription through antioxidant pathways (Ai et al., 2025; Ni et al., 2020).

Similarly, NOTUM is an endogenous secreted carboxylesterase that acts as another key negative feedback regulator. It inactivates Wnt ligands by removing palmitoleoyl modifications, thereby preventing Wnt ligands from binding to FZD receptors (Kakugawa et al., 2015). Pharmacological inhibition of NOTUM preserves the endogenous active pool of Wnt ligands. LP-922056 is a first-in-class, orally bioavailable NOTUM inhibitor with a thienopyrimidine scaffold. It has been structurally optimized to efficiently restore Wnt signaling and significantly increases cortical bone thickness and strength in preclinical murine models, while avoiding the broad toxicity caused by excessive intracellular Wnt hyperactivation (Brommage et al., 2019).

Notably, these extracellular targeted small molecules overcome the immunogenicity and administration burdens of monoclonal antibodies, retaining the specificity of targeting endogenous Wnt regulators. However, they still rely on continuous target occupancy to maintain efficacy, and thus do not fully transcend the inherent stoichiometric limitations of occupancy-driven pharmacology.

##### 3.1.2.2 Direct Wnt/β-catenin agonists and Wnt surrogates

Direct Wnt agonists initiate *de novo* canonical pathway activation by binding to core positive signaling components, independent of endogenous Wnt ligand activity or negative regulator inhibition. This class overcomes a critical limitation of indirect modulators: their inability to restore Wnt signaling in pathological states with severely suppressed endogenous ligand expression (e.g., advanced age-related bone loss).

Developing traditional small molecule agonists that directly bind to the extracellular domains of Frizzled (FZD) and LRP5/6 receptors has proven exceptionally difficult, as endogenous Wnt ligands rely on a post-translational lipid modification (palmitoleoylation) to engage the deep hydrophobic lipid-binding pocket of FZD, which poses an insurmountable obstacle for small-molecule drug discovery (Janda et al., 2012; Kahn, 2014). To bypass this “undruggable” lipid dependency, the field has undergone a major paradigm shift toward protein engineering, particularly the development of bispecific Wnt surrogates. These water-soluble, heterodimeric fusion proteins use independent binding domains to bridge FZD and LRP receptors, successfully phenocopying canonical Wnt signaling without requiring the hydrophobic lipid moieties of endogenous Wnts (Janda et al., 2017).

This breakthrough has profound implications for targeted therapy. Wnt surrogates can be modularly engineered to bind specific FZD subtypes, offering a rational strategy to achieve tissue restricted Wnt activation, and mitigate the systemic toxicity and tumorigenic risks of pan-Wnt overactivation. However, clinical translation of Wnt surrogates for osteoporosis remains hindered by severe pharmacological bottlenecks, including potential immunogenicity, high manufacturing costs, and insufficient targeted delivery into the dense bone matrix, and remains strictly in the preclinical evaluation phase.

Despite their potential, direct Wnt agonists face several translational barriers, including the narrow therapeutic window of systemic Wnt activation, limited FZD-subtype selectivity, and low bone-specific accumulation after systemic administration. Future rational design should focus on integrating bone-seeking chemical moieties with subtype-selective agonist scaffolds to achieve skeleton-restricted Wnt activation with minimal systemic exposure.

##### 3.1.2.3 GSK-3β inhibitors

Both synthetic small molecules and natural product derivatives have been explored as GSK-3β inhibitors, with a focus on overcoming the kinase selectivity and systemic toxicity pitfalls of first-generation agents.

The aminopyrimidine derivative CHIR-99021 serves as the gold-standard *in vitro* tool compound, with high kinome selectivity via dual hydrogen bond interactions with the kinase hinge domain (Ring et al., 2003). However, its poor *in vivo* metabolic stability, negligible oral bioavailability, and dose-limiting systemic toxicity restrict its utility exclusively to preclinical *in vitro* assays. Structural diversification efforts have yielded improved candidates: the indole derivative IM-12 is an ATP-competitive agent capable of maintaining *in vivo* osteogenic efficacy in ovariectomized (OVX) murine models without triggering overt systemic toxicity (Schmöle et al., 2010). Tideglusib, a non-ATP-competitive, irreversible inhibitor with a thiadiazolidinedione (TDZD) framework, provides a sustained blockade that may mitigate the rebound effects of conventional reversible occupancy-driven inhibitors (Domínguez et al., 2012). It has been evaluated in non-skeletal clinical settings, whereas its skeletal and dental repair potential remains mainly supported by preclinical evidence.

The prenylated flavonoid glycoside icariin, and its active aglycone metabolite icaritin, derived from Epimedium brevicornum, have demonstrated dual anabolic and anti-resorptive effects via GSK-3β inhibition and sclerostin suppression, with validated efficacy in OVX and GIOP murine models (Wei et al., 2020). However, their clinical translation is severely limited by extremely low native oral bioavailability, with ongoing efforts focused on bone-targeted nanoformulation optimization (Sheng et al., 2025).

Non-selective systemic modulation of the Wnt pathway carries severe skeletal safety risks. For example, PORCN inhibitors (e.g., ETC-159), which block Wnt ligand secretion for oncology indications, have been associated with dose-dependent bone loss and increased fracture risk in clinical trials, directly validating the critical role of tightly regulated Wnt signaling in skeletal homeostasis. This further highlights the necessity of bone-targeted, subtype-selective Wnt modulator design to avoid off-target skeletal toxicity.

While optimized GSK-3β inhibitors have improved selectivity and safety profiles compared to first-generation agents, most remain occupancy-driven molecules that require sustained systemic exposure, carrying inherent risks of off-target kinase inhibition and pleiotropic pathway dysregulation.

##### 3.1.2.4 Intracellular pathway modulators: SIK inhibition and β-catenin stabilization

Salt-inducible kinases (SIK2/SIK3) have been identified as critical intracellular hubs that orchestrate osteocyte responses to parathyroid hormone (PTH). YKL-05-099, a multi-kinase SIK inhibitor, acts as a small molecule PTH mimetic. It induces nuclear accumulation of HDAC4/5 to repress sclerostin (SOST) expression, while concurrently inhibiting bone resorption via off-target CSF1R blockade. Systemic administration of YKL-05-099 markedly promotes bone formation, and increases trabecular bone mass (Wein et al., 2016).

SKL2001 acts as a highly selective modulator that specifically disrupts the β-catenin–Axin protein-protein interaction (PPI). By directly binding the Armadillo repeat domain of β-catenin, it competitively displaces Axin, shielding β-catenin from ubiquitination and proteasomal degradation without affecting GSK-3β kinase activity (Gwak et al., 2011). This mechanistic precision circumvents the widespread off-target toxicity associated with pan-GSK-3β blockade, and has been validated to promote MSC osteogenic differentiation and accelerate bone defect repair *in vivo* (Liu et al., 2022; Sun et al., 2025). However, SKL2001 remains a classical occupancy-driven agent, with biological activity strictly dependent on sustained target binding.

#### 3.1.3 Clinical translation in osteoarthritis: lorecivivint (SM04690)

Lorecivivint, a small-molecule inhibitor of CLK2 and DYRK1A, modulates Wnt pathway activity through intranuclear kinase inhibition and is administered via localized intra-articular injection, thereby limiting systemic exposure and potentially reducing the toxicity associated with broad systemic Wnt modulation (Deshmukh et al., 2019). Although two pivotal single-dose phase III trials (OA-10 and OA-11) failed to meet their primary pain-reduction endpoints (Yazıcı et al., 2025a; Yazıcı et al., 2025b), a 3-year repeat-dose phase III extension study (OA-07) suggested preservation of medial joint space width, with more pronounced structural benefits in selected patient subgroups (Swearingen et al., 2024; Tambiah et al., 2025).

In summary, small-molecule Wnt modulators have addressed several limitations of conventional monoclonal antibodies and expanded the therapeutic scope of Wnt-targeted strategies. However, most remain occupancy-driven and require continuous systemic exposure, increasing the risks of off-target toxicity and narrow therapeutic windows. These limitations have prompted growing interest in targeted protein degradation and bone-selective epigenetic reprogramming.

### 3.2 RANKL/RANK/OPG axis inhibitors

The RANKL/RANK/OPG signaling network tightly coordinates the bone resorption phase of remodeling (Boyle et al., 2003). The anti-RANKL monoclonal antibody denosumab has clinically validated the therapeutic relevance of the NF-κB/NFATc1-activating cascade, but its strict reliance on occupancy-driven pharmacology creates substantial long-term limitations. In addition to the burdens of continuous injection, abrupt cessation of denosumab triggers a devastating rebound in osteoclast activity, frequently resulting in vertebral fractures (Kong et al., 2022; Lacey et al., 2012). To address these biologic-specific limitations, the field is increasingly prioritizing the development of titratable small molecule modulators targeting the RANKL interface, with a focus on overcoming the core limitations of occupancy-driven therapies.

#### 3.2.1 Small molecule PPI disruptors of the RANKL/RANK complex

Small molecule protein–protein interaction (PPI) disruptors offer translational advantages over large biologics: they enable oral administration, carry no immunogenicity risks, and allow tunable pharmacokinetic profiles through structure-based optimization. Unlike denosumab, which exerts only transient extracellular blockade, these small molecules can be chemically modified for bone-specific delivery to minimize systemic off-target effects.

Directly targeting the RANKL trimerization interface offers a strategic avenue to hinder RANK engagement, without depleting the available receptor pool. Among the developed candidates, S3-15 represents a critical mechanistic breakthrough. Through its dibenzylamine core, S3-15 anchors into a unique binding pocket structurally exclusive to soluble RANKL (sRANKL). This precise conformational targeting fundamentally uncouples bone resorption from systemic immune regulation, allowing it to halt ovariectomy-induced bone loss while completely sparing membrane-bound RANKL-mediated T and B cell functions (Huang et al., 2022).

Efforts have focused on optimizing the pharmacokinetic profiles of RANKL antagonists. The β-carboline derivative Y1693 achieves high-affinity binding to the RANK interaction interface on the RANKL trimer, with oral bioavailability and anti-resorptive efficacy, comparable to alendronate in OVX murine models (Yang et al., 2022). The 2-amino-3-N-phenylpropanamide derivative Compound 34 effectively targets the RANK-binding pocket and provides robust bone protection with excellent tolerability (Jiang et al., 2019). Collectively, these diverse small molecules shift the anti-resorptive paradigm away from global RANKL neutralization, proving that precise structural tuning can uncouple skeletal protection from systemic immunosuppression.

Despite these advances, these PPI disruptors still rely on continuous target occupancy to inhibit RANKL-RANK signaling, and do not fully resolve the risk of rebound.

#### 3.2.2 Transcriptional regulation of RANKL

Rather than strictly designing novel PPI disruptors, intercepting the upstream transcriptional regulation of RANKL provides a pragmatic avenue for drug repurposing. The legacy L-type calcium channel blocker verapamil illustrates the nuances of this approach. Historically, large meta-analyses concluded that standard antihypertensive regimens of calcium channel blockers (CCBs) yield a neutral effect on macroscopic fracture rates (Wiens et al., 2006). However, this apparent lack of efficacy largely stems from a pharmacokinetic mismatch: systemic cardiovascular dosing fails to reach the local microenvironmental threshold required for skeletal reprogramming.

When adequate localized concentrations are achieved, verapamil acts as a robust indirect suppressor of osteoclastogenesis. Specifically, it downregulates thioredoxin-interacting protein, which subsequently throttles the MAPK and NF-κB cascades necessary for RANKL-driven differentiation (Cao et al., 2025). Supported by recent retrospective cohort data correlating long-term verapamil exposure with preserved bone mineral density in postmenopausal women, redefining the localized pharmacology of such established agents offers a highly actionable path for osteoporosis intervention (Cao et al., 2025). Similar in their strategy, nutraceuticals such as the plant sterol β-sitosterol represent a class of non-directly osteoclastogenic compounds under active exploration. They exert indirect antiresorptive effects by attenuating cAMP/PKA and NF-κB signaling, thereby expanding the structural diversity of agents that intercept osteoclastogenic transcription (Guo et al., 2025).

In summary, small-molecule RANKL modulators and repurposed agents have addressed key limitations of denosumab, including oral bioavailability, reduced immunogenicity risk, and improved immune-sparing selectivity. However, these agents remain anchored in the occupancy-driven pharmacological framework, with efficacy strictly dependent on sustained target engagement. To permanently resolve the rebound osteoclastogenesis and long-term safety liabilities of conventional antiresorptives, event-driven modalities such as targeted protein degradation (PROTACs) represent the most transformative next-generation strategy, as detailed in the following section.

### 3.3 Microenvironment reprogramming: senolytics and SASP neutralizers

**FIGURE 3:**
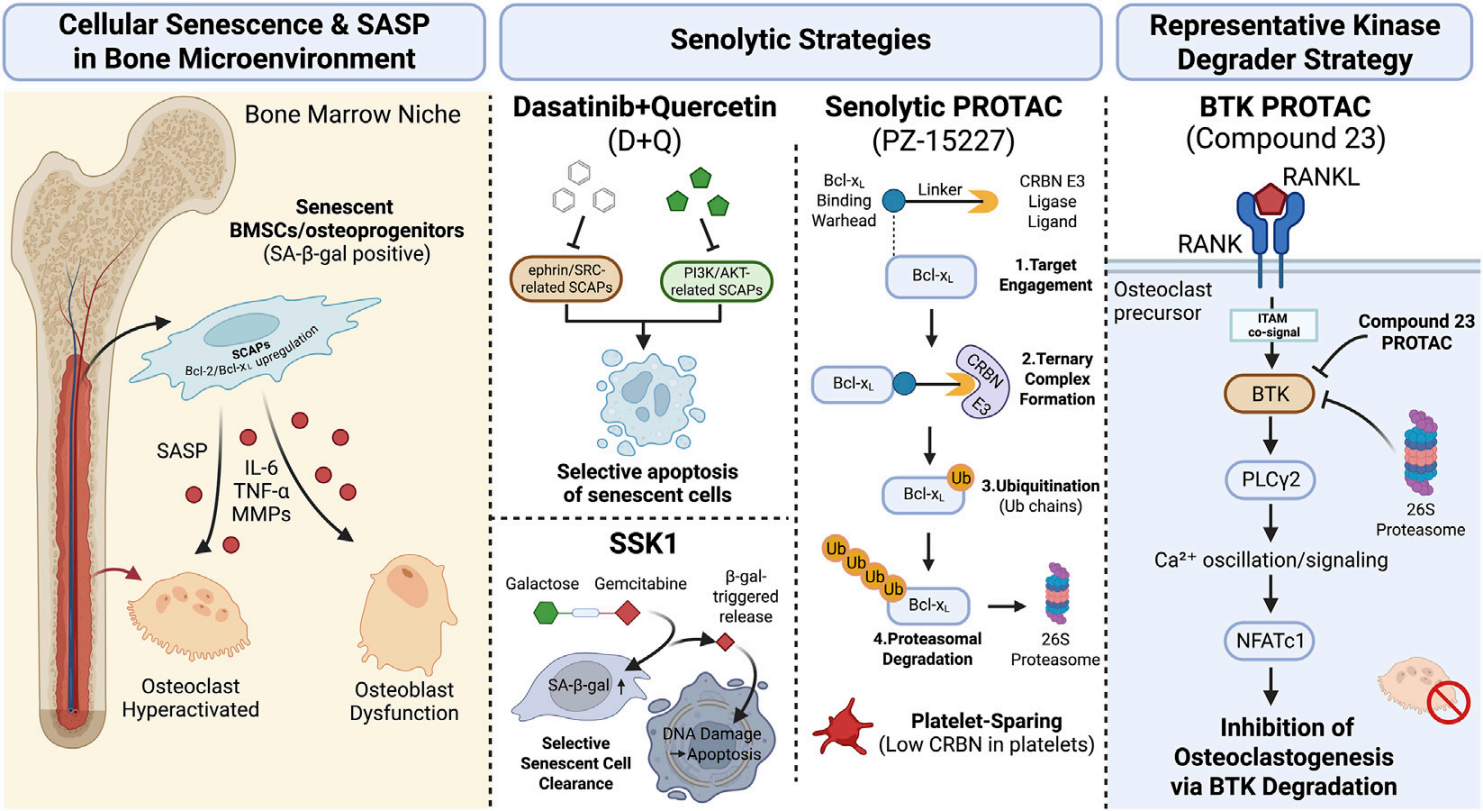
Cellular senescence and representative event-driven therapeutic strategies in the bone microenvironment. Senescent BMSCs and osteoprogenitors accumulate within the bone marrow niche and establish a SASP-rich microenvironment that promotes osteoclast activation and osteoblast dysfunction. Representative senolytic approaches include dasatinib plus quercetin (D + Q), which targets complementary senescent cell survival pathways, and the SA-β-gal-responsive prodrug SSK1, which releases gemcitabine selectively within senescent cells. PZ-15227 exemplifies a platelet-sparing Bcl-xL-degrading senolytic PROTAC, although its efficacy in bone-specific or bone marrow senescence models remains to be established. A representative kinase degrader strategy is illustrated by the BTK PROTAC compound 23, which suppresses osteoclastogenesis by catalytically depleting BTK and downregulating the downstream PLCγ2–Ca^2+^–NFATc1 signaling axis. Together, these approaches reflect the transition from reversible pathway inhibition to event-driven elimination of pathogenic cells or proteins within the diseased bone microenvironment.

To bypass the limitations of conventional occupancy-driven pharmacology, recent therapeutic strategies have pivoted toward rescuing the pathological skeletal microenvironment. At the core of this microenvironmental decay is cellular senescence. Accumulated senescent cells actively disrupt local homeostasis by releasing the senescence-associated secretory phenotype (SASP). The SASP disrupts bone remodeling by promoting osteoclast-mediated resorption while suppressing osteoblast differentiation (Farr et al., 2017; Khosla et al., 2020). Two primary strategies have been proposed to address this pathology: event-driven senolytics, which force the apoptosis of these aberrant cells, and occupancy-driven senomorphics (SASP neutralizers). These intervention strategies are summarized in Figure 3.

#### 3.3.1 Senolytics and natural flavonoids

The combination of dasatinib and quercetin (D + Q) established the foundational proof-of-concept for skeletal senolysis. The rationale behind this combination stems from the lineage-specific vulnerabilities of heterogeneous senescent pools. Because senescent cells rely on highly heterogeneous survival networks, single agents typically face severe apoptotic resistance. Mechanistically, clearing the senescent burden requires multiple survival networks at once. Dasatinib specifically handles the ephrin-dependent mesenchymal cells; however, the regimen strictly relies on quercetin to dismantle the PI3K/Bcl-2 pathways protecting the endothelial and immune compartments (Farr et al., 2017). The dramatic skeletal rejuvenation observed in murine models of trabecular and disc degeneration (Chaib et al., 2022; Childs et al., 2017; Novais et al., 2021) has notoriously failed to materialize in the clinic. A recent Phase II trial in postmenopausal women with osteoporosis showed limited overall skeletal benefit in the full cohort, with no significant reduction in CTx and only a transient, borderline increase in P1NP at 1 month (Farr et al., 2024). This sobering clinical reality dispels the notion of a universal senolytic ‘magic bullet’ and dictates a necessary pivot toward biomarker-stratified trial designs.

Subsequent optimization of the flavonoid backbone led to fisetin. Structurally, fisetin lacks the A-ring 5-hydroxyl group found in quercetin. This single deletion drastically increases the molecule’s lipophilicity and shifts its kinase affinity, driving a much stronger senolytic response within osteolineage cells (Zhu et al., 2017). Translating these cellular benefits, however, requires overcoming the compound’s dismal oral bioavailability. Formulating fisetin within PLGA nanoparticles resolves this primary pharmacokinetic barrier and reveals an expanded therapeutic profile. Rather than acting strictly as a senolytic clearing the microenvironment, this nano-formulation actively forces MSCs toward osteogenic lineage commitment and mineralization (Carbonare et al., 2022).

#### 3.3.2 Bcl-2 family inhibitors and bone-targeted prodrugs

The Bcl-2 anti-apoptotic network presents a target for senescent clearance (Zhu et al., 2015). Navitoclax (ABT-263) exposes the limitations of systemic senolysis. In skeletal applications, Navitoclax directly depletes osteoprogenitor pools and accelerates trabecular bone loss in aged murine models (Sharma et al., 2020). Combined with unavoidable dose-limiting thrombocytopenia (Chang et al., 2015), this non-selective Bcl-2 blockade is non-viable for osteoporosis management.

To bypass these toxicities, targeted prodrug platforms have emerged to restrict payload activation exclusively to the pathological niche, utilizing specific enzymatic signatures like senescence-associated β-galactosidase (SA-β-gal). Exploiting this biomarker, researchers engineered SSK1, a prodrug that conjugates a galactose moiety to a cytotoxic payload. For example, the SSK1 compound utilizes a galactose moiety to structurally cage the cytotoxic agent gemcitabine. This ensures the drug remains systemically inactive until it encounters the high SA-β-gal concentrations specific to senescent cells. Upon local enzymatic cleavage, gemcitabine is unmasked, driving targeted apoptosis without the off-tissue destruction of platelets and healthy progenitors seen with Navitoclax (Cai et al., 2020). Pending direct *in vivo* validation in bone, this SA-β-gal-responsive caging establishes a highly translatable proof-of-concept for skeletal precision therapy. Future medicinal chemistry innovations are exploring dual-targeted senolytics to achieve highly specific enrichment within the bone matrix.

#### 3.3.3 SASP neutralizers

An alternative strategy employs senomorphics to functionally disarm the SASP. Because the SASP is predominantly regulated by NF-κB and JAK/STAT transcriptional networks, small-molecule interventions may attenuate the inflammatory microenvironment (Chien et al., 2011; Xu et al., 2015). For example, the synthetic triterpenoid bardoxolone methyl (CDDO-Me) acts as a dual modulator, suppressing NF-κB while activating Nrf2. JAK1/2 inhibitors built on pyrrolopyrimidine scaffolds potently restrict the local accumulation of IL-6 and TNF-α. By starving osteoclasts of these pro-inflammatory cues, these agents have gained significant traction in models of age-related bone loss, tumor osteolysis, and rheumatoid arthritis (Khosla et al., 2020).

Epigenetic regulation provides another senomorphic axis. SIRT1, an NAD + -dependent deacetylase, is a known SASP suppressor. Because of the profound metabolic instability, researchers developed SRT2104 as a SIRT1 activator. Systemic SRT2104 successfully maintains bone mass and cortical biomechanics in aged murine models, establishing SIRT1 modulation as a highly tractable pharmacological approach for reprogramming the skeletal niche (Mercken et al., 2014).

#### 3.3.4 Challenges and the transition to targeted protein degradation

The clinical viability of microenvironment reprogramming is ultimately bottlenecked by the heterogeneity of cellular senescence. Because of the drastically different Senescent Cell Anti-Apoptotic Pathways (SCAPs), it is biologically implausible to achieve broad-spectrum senolysis with a single agent (Gasek et al., 2021). To bypass these structural roadblocks, the field is increasingly transitioning toward an event-driven framework via Targeted Protein Degradation. By co-opting the cellular ubiquitin-proteasome system, PROTACs provide a highly modular solution to permanently eliminate pathogenic targets that classical small molecules cannot effectively drug, offering a critical workaround for precision skeletal therapies (detailed in Section 3.4).

### 3.4 PROTACs in bone pharmacology

Translating the event-driven paradigm into skeletal therapeutics relies heavily on the unique heterobifunctional architecture of PROTACs. By chemically linking a target-binding ligand to an E3 ubiquitin ligase recruiter (such as CRBN or VHL) via a tailored linker, these molecules induce the formation of a ternary complex. This structural proximity forces the polyubiquitination and subsequent proteasomal degradation of the target protein (Sakamoto et al., 2001). This catalytic elimination offers profound advantages. It abrogates both the catalytic kinase and scaffolding functions of the target, overcomes drug resistance, and sustains target depletion after the drug has cleared from circulation (Békés et al., 2022).

#### 3.4.1 BTK-PROTACs: validated degraders in inflammatory bone loss

Bruton’s tyrosine kinase (BTK) is a critical intracellular downstream effector in the RANKL-RANK axis, essential for osteoclast differentiation and inflammatory signaling. While conventional BTK inhibitors exist, their efficacy in bone protection is limited by incomplete signaling blockade. Recently, the application of PROTACs has successfully broken this bottleneck. Researchers developed novel BTK PROTACs using spebrutinib-derived ligands (Huang et al., 2024). Compound 23 emerged as a first-in-class, highly potent BTK degrader. It exhibits outstanding degradation potency (DC_50_ = 1.29 nM at 4 h) and rapid clearance kinetics via a CRBN-dependent mechanism. In murine models of periodontitis, Compound 23 treatment robustly attenuated osteoclastogenesis and prevented alveolar bone loss. Beyond osteoporosis, BTK degraders are currently entering clinical trials for autoimmune diseases like rheumatoid arthritis, expanding the application of targeted protein degradation to broad inflammatory skeletal pathologies.

#### 3.4.2 Senolytic PROTACs: precision microenvironment reprogramming

Targeting Bcl-xL for senolysis is traditionally limited by severe on-target thrombocytopenia. PZ-15227 was designed to reduce this toxicity by recruiting CRBN, an E3 ligase that is less abundant in platelets, thereby favoring Bcl-xL degradation in senescent cells over platelets (He et al., 2020). In preclinical systems, this design enhanced senolytic activity while reducing platelet toxicity. However, whether PZ-15227 can selectively clear senescent cells within the bone marrow microenvironment and improve skeletal outcomes has not yet been directly established. Therefore, PZ-15227 should be regarded as a platelet-sparing senolytic PROTAC with potential relevance to skeletal niche reprogramming rather than as a bone-validated therapeutic strategy.

#### 3.4.3 Expanding the target space

Although PROTACs are effective at degrading intracellular effectors, such as BTK and survival proteins, a major challenge in skeletal pharmacology is the targeted degradation of key transmembrane drivers, such as RANK, to address rebound bone loss after denosumab discontinuation. Classical cytosolic PROTACs can engage the intracellular domains of transmembrane proteins in some disease models but remain ineffective against fully extracellular targets. Furthermore, no RANK-specific PROTAC has been successfully translated into bone metabolism research.

To address this limitation and achieve the complete elimination of extracellular signaling, the chemical biology field is rapidly developing Lysosome-Targeting Chimeras (LYTACs). LYTACs capture extracellular or membrane-bound proteins via target-specific ligands and shuttle them to the lysosome for degradation through cell-surface receptor-mediated endocytosis (Banik et al., 2020). Conceptually, lysosomal depletion of RANK could attenuate RANK-dependent osteoclastogenic signaling and may provide a future strategy for addressing post-denosumab rebound. However, no RANK-targeted LYTAC has yet been reported in bone metabolism models. Its feasibility will depend on achieving sufficient skeletal delivery, receptor internalization, and lysosomal trafficking in osteoclast-lineage cells. In addition, the relatively large molecular size of current LYTAC platforms may limit penetration into the bone remodeling microenvironment. Accordingly, RANK-targeted LYTACs should currently be regarded as a forward-looking chemical-biology concept rather than a validated solution to denosumab-withdrawal rebound.

Additionally, osteoblast and osteocyte apoptosis in GIOP may represent a future application area for targeted degradation (Weinstein, 2011). Functional glucocorticoid receptor PROTACs, such as KH-103, have been developed in non-skeletal neuronal models to counteract excessive glucocorticoid receptor activation (Gazorpak et al., 2023). However, their skeletal delivery, cell-type selectivity, pharmacodynamic effects, and efficacy in GIOP have not been established. Bone-targeted GR degraders should therefore be regarded as a conceptual research direction requiring substantial preclinical validation rather than as an immediately translatable therapeutic strategy.

#### 3.4.4 Challenges and future directions for degraders in bone

Despite offering unprecedented therapeutic opportunities, targeted protein degradation faces specific translational hurdles in bone pharmacology.

##### 3.4.4.1 Skeletal selectivity via bone-targeted chemical design and linker optimization

Ensuring that PROTACs preferentially degrade targets in bone while sparing peripheral tissues is critical. To achieve this, researchers are engineering “Bone-Targeted PROTACs” by conjugating bisphosphonate functional groups directly onto the PROTAC linker. This modification endows the massive heterobifunctional molecule with high affinity for hydroxyapatite, achieving specific enrichment within the bone matrix and minimizing systemic exposure. This bisphosphonate-mediated skeletal enrichment principle has recently been validated in more complex heterobifunctional molecules such as BT-Amide (Wang et al., 2024). As a bisphosphonate-conjugated Pyk2 inhibitor, BT-Amide serves as a crucial proof-of-concept and provides a foundational chemical blueprint for developing next-generation bone-targeted PROTACs.

Beyond tissue-specific enrichment, the intracellular catalytic efficiency of PROTACs is strictly dictated by the structural topology of the chemical linker. The linker is not a passive tether but a crucial spatial regulator that determines the orientation and effective molarity between the target protein and the E3 ligase. A key optimization challenge lies in balancing the trade-off between flexibility and rigidity. While flexible PEG chains mitigate steric clashes, excessive length increases conformational entropy, thereby reducing the probability of productive ternary complex formation. Conversely, introducing rigid motifs can pre-organize the complex, yet a linker that is too short inevitably leads to steric hindrance.

Furthermore, PROTAC efficacy is profoundly limited by the stoichiometric “hook effect”—a phenomenon where elevated drug concentrations independently saturate both the target and the E3 ligase, driving the equilibrium toward inactive binary complexes rather than the productive ternary complex. Overcoming this auto-inhibition demands meticulous structure-activity relationship tuning. For example, during the optimization of BTK degrader Compound 23, the linker needed to be incrementally extended from PEG3 to PEG5 to relieve steric tension, ultimately achieving low-nanomolar degradation potency without triggering a premature hook effect (Huang et al., 2024).

Crucially, this stoichiometric vulnerability becomes a defining bottleneck for bone-targeted PROTACs. The incorporation of bone-homing moieties like bisphosphonates forces a massive local accumulation of the drug at bone remodeling surfaces. While this extreme spatial enrichment is the exact goal of targeted delivery, it inadvertently primes the local microenvironment for the hook effect. Consequently, the successful development of skeleton-restricted degraders demands exceptionally rigorous SAR optimization of linker architecture and precise dosing strategies to ensure that localized drug pooling drives target degradation rather than self-antagonism.

##### 3.4.4.2 Oral bioavailability optimization

PROTACs typically possess high molecular weights and heavily violate Lipinski’s ‘rule of five,’ posing inherent challenges for oral administration. While intermittent intravenous dosing represents the mainstream route for PROTACs in current clinical trials, recent medicinal chemistry advances demonstrate that optimizing the lipophilicity, intramolecular hydrogen bonding, and rigidity of the linker can significantly improve oral bioavailability—even for structurally complex, bisphosphonate-conjugated bone-targeted derivatives (Pike et al., 2020; Wang et al., 2024). For chronic progressive diseases such as senile and glucocorticoid-induced osteoporosis, successfully optimizing oral administration remains a mandatory milestone for the long-term clinical translation of bone-targeted PROTACs.

##### 3.4.4.3 E3 ligase selection for skeletal tissue specificity

The catalytic efficacy of PROTACs strictly depends on the basal expression levels of the recruited E3 ligase (e.g., CRBN or VHL) within the target cells. Developing degraders that hijack E3 ligases with highly restricted tissue expression is currently recognized as the ultimate strategy to overcome off-target toxicities. For bone pharmacology, comprehensive mapping of E3 ligase expression dynamics during osteoblast and osteoclast differentiation will be fundamentally necessary to tailor PROTACs for precise skeletal pathologies without affecting peripheral organs (Békés et al., 2022).

Collectively, PROTACs and emerging targeted degradation technologies effectively bypass the limitations of traditional occupancy-driven skeletal therapeutics. By integrating event-driven target elimination with bone-specific chemical design, these modalities offer a promising framework for addressing key clinical challenges in osteoporosis and related skeletal disorders.

### 3.5 Direct metabolic modulators: targeting the lactate-epigenetic axis

Bridging the gap between mechanistic metabolic insights and clinical utility requires medicinal chemistry to intercept specific checkpoints governing both lactate flux and its downstream epigenetic translation. Rather than relying on global monocarboxylate transport blockade, achieving true skeletal-restricted reprogramming will increasingly depend on the precise modulation of bone-specific epigenetic “writers.”

#### 3.5.1 Isoform-selective disruption of lactate flux

To resolve the paradoxical challenge of inhibiting osteoclastic lactate efflux without impairing the essential uptake requirements of osteoblasts, research has pivoted toward isoform-selective intervention. Unlike generic monocarboxylic acid transport inhibitors (e.g., syrosingopine) (Benjamin et al., 2018) or MCT1-specific inhibitors (e.g., AZD3965) (Beloueche-Babari et al., 2017; Curtis et al., 2017), these agents do not carry a high risk of perturbing lactate homeostasis in osteoblasts or worsening epigenetic silencing. MCT4-selective inhibitors, such as the VB124 scaffold, offer a conceptual strategy for limiting osteoclast-associated lactate efflux while potentially sparing MCT1-dependent lactate handling in osteoblast-lineage cells (Fang et al., 2022; Nishioku et al., 2023). By driving terminal intracellular lactic acidosis specifically within active osteoclasts, these agents may limit osteoclast-associated resorptive activity while potentially sparing MCT1-dependent lactate handling in osteoblast-lineage cells (Wu J. et al., 2023; Xu et al., 2026). The theoretical contraindication of broad MCT blockade in osteoporosis may be partially circumvented, illustrating a refined metabolic intervention. Although this approach is mechanistically attractive, direct validation in osteoporosis-specific models remains limited.

#### 3.5.2 Epigenetic rejuvenation: from p300 to the AARS1/2 frontier

Moving beyond metabolite transport, pharmacologically modulating the “writers” that catalyze histone lactylation presents a direct route to unlock silenced osteogenic programs in aged skeletal tissues.

##### 3.5.2.1 p300/CBP: a bivalent rheostat with translational risks

The p300/CBP axis currently serves as the most extensively characterized, albeit highly pleiotropic, writer for these epigenetic marks. Catalytic inhibitors such as A-485 have proven effective at erasing pathological lactylation signatures in osteoarthritis models (Lasko et al., 2017; Xu et al., 2026). However, applying such global acetyltransferase inhibitors systemically for osteoporosis introduces unacceptable oncogenic liabilities. To circumvent this, the field is pivoting toward “event-driven” p300-PROTACs, building on early degraders like dCBP-1 (Vannam et al., 2021), to selectively eliminate dysfunctional p300 subpopulations tethered to the SASP without abolishing basal enzyme function.

##### 3.5.2.2 AARS1/2: closing the logic loop for precision bone therapy

As delineated in Section 2.2, identifying AARS1 and AARS2 as non-canonical lysine lactyltransferases fundamentally reframes our understanding of skeletal mechanobiology (Li et al., 2024; Zong et al., 2024). Unlike the broadly active p300, AARS enzymes act as specialized metabolic sensors that link local lactate fluctuations directly to downstream target modifications, including H3K18la (Hong et al., 2025). Despite robust validation of AARS1-mediated H3K18 lactylation in driving aberrant cellular states, a glaring absence of bone-optimized small-molecule inhibitors persists. Existing tool compounds predominantly function as competitive inhibitors at the aminoacylation site, a mechanism flawed by its inevitable interference with essential protein synthesis. Achieving the therapeutic index required for chronic bone diseases will necessitate the design of allosteric modulators capable of uncoupling the lactyltransferase activity from the enzyme’s canonical translational duties. At present, no bone-optimized AARS1/2 inhibitor or degrader has been established. Future studies should first determine whether the non-canonical lactyltransferase activity of AARS1/2 can be pharmacologically separated from their essential aminoacylation functions. Bone-targeted delivery using established ligands, such as the CH6 aptamer or acidic oligopeptides, could subsequently be explored to improve skeletal selectivity (Liang et al., 2015; Zhang et al., 2012). However, the feasibility, cell-type specificity, and therapeutic window of such approaches remain unknown. Accordingly, allosteric AARS1/2 modulators and AARS-targeted PROTACs should currently be regarded as forward-looking research concepts rather than experimentally validated skeletal therapies.

## 4 Clinical translation and delivery challenges

### 4.1 Clinical translation

The translational pipeline for novel skeletal therapeutics is witnessing a profound transition from occupancy-driven biologics to rationally designed small molecules. Given the systemic safety constraints and structural limitations of existing macromolecular therapies, there is an urgent clinical void for tissue-selective, mechanism-based modulators. To further strengthen the clinical translation perspective, Table 2 stratifies representative interventions according to their evidence level, including approved bone therapies, investigational bone or joint candidates, senolytic strategies with human evidence, and repurposing candidates previously evaluated in non-skeletal clinical settings. The development of Lorecivivint (SM04690) illustrates a highly promising path. By targeting CLK2 and DYRK1A via localized intra-articular delivery, it limits systemic exposure and may reduce the toxicity associated with broad systemic Wnt modulation (Deshmukh et al., 2019). Its efficacy relies on dampening β-catenin hyperactivation to arrest OA progression. This strategy of localized, precision ‘fine-tuning’ respects the compartmentalized nature of joint homeostasis, providing a useful model for the design of next-generation disease-modifying osteoarthritis drugs. The two pivotal single-dose phase III trials (OA-10 and OA-11) did not meet their primary pain-reduction endpoints (Yazıcı et al., c2025a; 2025b), whereas a 3-year repeat-dose phase III extension study (OA-07) suggested structural benefits. Treatment was associated with a trend toward preservation of medial joint space width across the overall study population, with statistically significant structural protection observed in selected patients (Swearingen et al., 2024; Tambiah et al., 2025).

**TABLE 2:** Representative clinical evidence and translational status of therapies and candidates relevant to bone metabolism-related disorders. This table summarizes approved bone therapies, investigational bone or joint candidates, senolytic strategies with human evidence, and repurposing candidates with prior non-skeletal clinical evaluation.

Compound/strategy	Target/modality	Evidence category/clinical stage	Indication	Key outcomes	Current status/translational implication
Teriparatide	PTH1R agonist; anabolic therapy	Approved bone therapy; pivotal clinical evidence (Neer et al., 2001)	Postmenopausal or high-fracture-risk osteoporosis	Reduced vertebral and nonvertebral fractures; anabolic benchmark	Approved clinical therapy; comparator for next-generation anabolic agents
Denosumab	Anti-RANKL monoclonal antibody; antiresorptive therapy	Approved bone therapy; established clinical use with documented discontinuation-related rebound risk (Kim et al., 2022; Kong et al., 2022)	Postmenopausal osteoporosis; GIOP and therapy-associated bone loss	Effective antiresorptive therapy, but discontinuation can trigger rebound bone turnover and vertebral fractures	Approved therapy; clinically validates RANKL blockade but exposes the rebound limitation of occupancy-driven inhibition
Romosozumab	Anti-sclerostin monoclonal antibody; Wnt-pathway anabolic therapy	Approved bone therapy; phase III FRAME and ARCH evidence (Cosman et al., 2016; Saag et al., 2017)	Postmenopausal osteoporosis at high fracture risk	Improved BMD and reduced vertebral fracture risk; cardiovascular warning limits patient selection and treatment duration	Approved anabolic therapy; validates Wnt activation and motivates safer, more selective Wnt modulators
Lorecivivint/SM04690	CLK2/DYRK1A inhibitor; localized Wnt-pathway modulator	Investigational bone/joint candidate; phase III OA-10 and OA-11 trials and extension studies (Yazıcı et al., 2025a, Yazıcı et al., 2025b; Swearingen et al., 2024; Tambiah et al., 2025)	Knee osteoarthritis	Single-dose trials did not meet primary pain endpoints; repeat-dose/extension analyses suggested structural benefit in selected populations	Investigational; represents localized Wnt modulation for joint structural protection
Dasatinib + quercetin (D + Q)	Senolytic combination targeting complementary SCAP networks	Investigational senolytic strategy; Phase II randomized clinical trial (Farr et al., 2024)	Postmenopausal women with skeletal aging/osteoporosis-related risk	Limited overall skeletal benefit; exploratory signal in participants with higher senescent-cell burden	Investigational senolytic strategy; supports biomarker-stratified trial design
Tideglusib	GSK-3β inhibitor; Wnt/β-catenin activator	Non-skeletal clinical exposure and repurposing candidate; skeletal and dental evidence remains mainly preclinical (Domínguez et al., 2012)	Potential dental repair and bone-defect regeneration	Preclinical regenerative potential; bone-specific clinical efficacy has not been established	Repurposing candidate; should not be interpreted as an established bone clinical therapy

Evidence categories: “Approved bone therapy” indicates a therapy approved for a skeletal indication; “Investigational bone/joint candidate” indicates an agent evaluated in bone- or joint-related clinical trials; “Investigational senolytic strategy” indicates a human-tested intervention that is not approved for a skeletal indication; and “Non-skeletal clinical exposure” indicates prior human evaluation in other indications, with skeletal evidence remaining primarily preclinical.

Abbreviations: BMD, bone mineral density; GIOP, glucocorticoid-induced osteoporosis; OA, osteoarthritis; PTH1R, parathyroid hormone receptor 1; RANKL, receptor activator of nuclear factor κB ligand; SCAP, senescent-cell anti-apoptotic pathway.

In the context of tumor-induced bone destruction, multi-modal interventions are required to restore the decoupled skeletal microenvironment. The DKK1 inhibitor WAY-262611, for instance, has expanded from an osteoporosis-oriented candidate to a preclinical anti-metastatic strategy. DKK1 inhibition may restore local osteoblastogenesis while disrupting pathological osteoblast–osteoclast coupling that contributes to osteosarcoma and breast cancer bone metastasis (Tal et al., 2025). Concurrently, microenvironment-reprogramming strategies have provided an early clinical test case for senolytic therapy in osteoporosis, such as the senolytic D + Q regimen (Farr et al., 2024).

### 4.2 Chemical strategies for skeletal delivery

Effective skeletal delivery remains a formidable hurdle for most novel chemical entities. To prevent systemic off-target effects, and overcome the poor oral bioavailability inherent to complex molecules, medicinal chemists are actively developing advanced delivery platforms.

#### 4.2.1 Diversifying bone-targeting chemistries

Selective accumulation at sites of bone remodeling is critical to reduce systemic toxicity from potent agents such as PROTACs and Wnt agonists. While conjugating active pharmacophores to bisphosphonates remains a benchmark strategy due to their exceptional affinity for hydroxyapatite, the permanent skeletal retention of BPs poses long-term clinical risks, including adynamic bone disease and osteonecrosis of the jaw (ONJ).

To address these drawbacks, researchers have expanded the range of bone-targeting chemistries. Acidic oligopeptides, such as aspartic acid octapeptides (Asp8), offer a highly translatable, biodegradable alternative. These peptides coordinate with calcium ions on the HA surface via multidentate bonds. Crucially, unlike BPs, peptide linkers undergo endogenous proteolytic cleavage after delivering their payload, ensuring complete metabolic clearance without cumulative toxicity (Zhang et al., 2012).

Advancing beyond passive “matrix targeting,” the field is pioneering “cell-specific targeting” using nucleic acid aptamers. For instance, the CH6 aptamer has been chemically engineered to bind exclusively to cell-surface biomarkers on active osteoblasts (Liang et al., 2015). By conjugating payloads to such aptamers, therapeutics can bypass the calcified matrix entirely and directly enrich within the cellular osteogenic niche, representing the ultimate evolutionary step in precision skeletal delivery.

#### 4.2.2 Nanoscale formulation optimization

Lipid-based nanoparticles (LNPs) and polymeric micelles are being engineered to alter the pharmacokinetic profiles of poorly water-soluble natural modulators (e.g., icariin and resveratrol). Rather than relying on direct portal vein absorption, these lipophilic nanocarriers exploit the intestinal lymphatic transport system. Specifically, the formulated compounds are packaged into chylomicrons within enterocytes and secreted directly into the lymphatic circulation. This route avoids hepatic first-pass metabolism, reducing premature degradation and markedly improving oral bioavailability and bone tissue exposure (Trevaskis et al., 2007).

Beyond improving systemic pharmacokinetics, locally retained or implant-integrated nanomaterials can directly reprogram diseased osteochondral and peri-implant microenvironments. Engineered MgO nanoparticles incorporated into PLGA microspheres enabled sustained Mg^2+^ delivery and coordinated protection of cartilage and subchondral bone in rat osteoarthritis models, with the cartilage–bone interaction primarily linked to PI3K/AKT signaling (Zheng et al., 2024). A reactive oxygen species-responsive hydrogel containing Cu^2+^-chelated epigallocatechin gallate nanoparticles and nanohydroxyapatite suppressed ferroptosis in bone marrow mesenchymal stem cells and promoted vascularized bone regeneration in a rat critical-sized defect model (Li et al., 2026). Extending this concept from injectable carriers to implant-integrated materials, ceria nanozyme-engineered ultra-high-molecular-weight polyethylene liners simultaneously reduced wear-particle generation and attenuated particle-induced macrophage inflammation and osteoclastic bone resorption (Liu et al., 2026). Together, these studies illustrate how nanoscale design can combine sustained local delivery, microenvironmental regulation, and structural integration to broaden skeletal therapeutic strategies beyond conventional drug carriers.

## 5 Future perspectives

The future of skeletal pharmacology lies in precision medicine, leveraging advances in computational chemistry, biomarker profiling, and rational combination strategies.

### 5.1 Biomarker-guided precision therapy

The shift toward event-driven therapeutics has made robust biomarkers essential for patient stratification and treatment response monitoring. Traditional bone turnover markers—including amino-terminal propeptide of type I procollagen (P1NP) and C-terminal cross-linking telopeptide of type I collagen (CTX)—remain useful, but the quantification of circulating senescence-related biomarkers has become an increasingly promising strategy for guiding senolytic therapy. These biomarkers include SA-β-gal-positive extracellular vesicles, key SASP factors (such as IL-6, TNF-α, and MMP-3), and cyclin-dependent kinase inhibitors (p16INK4a and p21CIP1) in peripheral blood mononuclear cells. A major barrier to clinical translation, however, is the lack of skeletal cell-specific senescence biomarkers. Systemic SASP levels are often confounded by age-related comorbidities like chronic inflammation, diabetes, and cardiovascular disease, making it difficult to link these systemic markers directly to skeletal senescence.

### 5.2 AI-driven chemical discovery and rational design

In bone therapeutics, artificial intelligence (AI)-driven chemical discovery has surmounted the principal limitations of conventional high-throughput screening by furnishing viable strategies for historically “undruggable” targets that have long impeded skeletal drug development. As elaborated in Section 3.2.1, the flexible, conformationally dynamic extracellular domain of the RANK receptor lacks deep hydrophobic pockets requisite for high-affinity small-molecule binding, rendering it recalcitrant to traditional rational design approaches (Liu et al., 2010; Scott et al., 2016). To overcome this, AI-powered structural prediction platforms are revolutionizing target engagement. For instance, advanced computational docking and structure-based virtual screening have already facilitated the discovery of novel small-molecule inhibitors (such as S3-15 and Compound 34) that successfully block the RANKL-RANK interaction interface (Huang et al., 2022; Jiang et al., 2019). Building on these milestones, the recent deployment of AlphaFold 3—which accurately predicts full biomolecular complexes including protein-ligand interactions—now enables the high-fidelity delineation of transient cryptic allosteric sites within the highly flexible RANK extracellular domain. This technological leap furnishes a robust, experimentally actionable rationale for the *de novo* design of previously ‘undruggable’ skeletal targets (Abramson et al., 2024; Jumper et al., 2021).

In the realm of bone-targeted proteolysis-targeting chimeras—the pivotal innovative modality central to this review—generative graph neural networks (GNNs) and all-atom molecular dynamics simulations are now routinely employed to quantitatively model the “hook effect” and predict the impact of linker length, rigidity, and chemical topology on the stability of the target-PROTAC-E3 ligase ternary complex. Looking forward, the integration of mechanism-informed computational design represents a major leap for skeletal PROTAC development. As highlighted by the BTK and Bcl-xL degraders discussed earlier (Section 3.4), this rational approach effectively bypasses the most notorious bottleneck in targeted degradation: the empirical, trial-and-error synthesis of massive linker libraries. By shifting from serendipitous discovery to predictive modeling, future development cycles can be dramatically accelerated (Békés et al., 2022; Huang et al., 2024). Artificial intelligence algorithms are harnessed to refine the bone-homing attributes of targeted conjugates and nanoformulations, affording high-fidelity predictions of hydroxyapatite-binding affinities for bisphosphonate, acidic oligopeptide, and aptamer-conjugated modifications, redressing the delivery impediments delineated in Section 4.2.

### 5.3 Spatiotemporal integration and “induction-maintenance” regimens

The clinical translation of emerging skeletal therapeutics may ultimately require mechanism-based combination regimens rather than empirical simultaneous co-administration. Inspired by the anabolic-window concept associated with romosozumab, a hypothetical sequential strategy of “niche-clearing” followed by “anabolic-induction” could be explored. In principle, reducing senescent-cell burden and SASP activity before administering an osteoanabolic agent may improve the responsiveness of osteoprogenitors. However, this concept has not yet been experimentally validated.

As one possible implementation, a short course of a platelet-sparing senolytic PROTAC, such as PZ-15227 or a future bone marrow-targeted derivative, could be followed by an osteoblast-targeted Wnt-modulating strategy, such as an engineered FZD-subtype-selective Wnt surrogate or an SKL2001 conjugate. If validated, this sequence might reduce the suppressive influence of SASP-associated cytokines on osteoanabolic responses and limit cumulative toxicity through intermittent dosing. Nevertheless, its feasibility will depend on demonstrating skeletal targeting, optimal dosing intervals, safety, and appropriate treatment sequencing, particularly in light of the limited overall benefit observed in the Phase II D + Q trial (Farr et al., 2024).

## 6 Conclusion

Skeletal pharmacology is undergoing a profound and lasting paradigm shift, moving from symptomatic inhibition to mechanistic reprogramming of the bone microenvironment. This shift directly addresses the unmet clinical needs. Current therapies cannot reverse the progressive bone loss caused by osteoblast apoptosis, cellular senescence, and breakdown of the bone marrow niche. The emerging agents, including Wnt pathway modulators, small molecule RANKL PPI inhibitors, bone-targeted senolytics, PROTAC degraders, and metabolic-epigenetic regulators, have shown considerable therapeutic promise in preclinical settings. Several critical obstacles still limit clinical translation, including the spatial limitations of bone-targeted delivery and the marked heterogeneity of the senescence-associated secretory phenotype (SASP) across skeletal cell lineages and patient populations. Moving beyond traditional single-target drug discovery toward targeted, patient-centered strategies will therefore be necessary.

Molecular biomarkers may help stratify patient populations and guide treatment selection. Future strategies should combine spatiotemporally controlled delivery with targeted protein degradation and local metabolic–epigenetic modulation, while using AI-assisted molecular design to address skeletal proteins that have long been considered undruggable.
